# Ants in Africa

**Published:** 1892-01

**Authors:** 


### ANTS IN AFRICA.

Silently, deadly and irresistibly move these battalions ; out of the forest, down, into, across and up the ditch, through the boma (wood stockade), across the square, and into every nook and cranny conceivable they swarmed. The first notice (they generally came at night) would be a loud yell from some of the men "Look out ! Siafu !" There would be no more sleep that night. After experience gained, says the *Nineteenth Century*, we found it the best plan to clear out of our houses, rush into the square and build rings of fire around us. To put on one's clothes was to get bitten by dozens all over one's body, unless they had been first thoroughly smoked over a fire. Every now and then yells and curses told how a lazy one had got caught in his bunk. The walls of the huts, the roofs and floors, were simply one seething mass of struggling ants. They were after the cockroaches, mice and insects that had taken up their abode in the roofs. Now and then squeaks of young mice told their story. As fast as the ants found their load, generally a cockroach, they would make off down the hill in long line. Luckily they never touched our granaries; they seemed to prefer animal food. Toward morning there would be only a few lost ones, aimlessly tearing about, apparently looking for the main body which had just decamped. Usually these raids on us were made after a rainstorm; many of them came into the fort already staggering under loads; these appeared to wander about till the others were ready. Next day not a cockroach could be found in the place, so that the ants did us a service in ridding us of these pests. The rats had decamped also, and did not return for some days. We have seen outside the forts, armies of red ants two and a half days long; i. e., they would take two and a half days passing a given spot. During the day the march would be incessant, every one marching at his very best; toward night they would huddle in a seething mass, and if disturbed scatter in all directions. The width of the stream of ants would be about two inches generally. On the flanks of this were the soldiers, fully twice the length of the workers. On our approach these big chaps would run out and up our legs like lightning. No birds, but of one sort, seemed to trouble them; these were little fellows about as big as sparrows, and of a dull gray color.

